# Coronavirus Infection of the Central Nervous System: Animal Models in the Time of COVID-19

**DOI:** 10.3389/fvets.2020.584673

**Published:** 2020-10-23

**Authors:** Peter J. Dickinson

**Affiliations:** Department of Surgical and Radiological Sciences, School of Veterinary Medicine, University of California, Davis, Davis, CA, United States

**Keywords:** GS-441524, remdesivir, SARS-CoV-2, feline infectious peritonitis (FIP), treatment

## Abstract

Naturally occurring coronaviral infections have been studied for several decades in the context of companion and production animals, and central nervous system involvement is a common finding, particularly in cats with feline infectious peritonitis (FIP). These companion and production animal coronaviruses have many similarities to recent human pandemic-associated coronaviruses such as SARS-CoV, MERS-CoV, and SARS-CoV2 (COVID-19). Neurological involvement is being increasingly recognized as an important clinical presentation in human COVID-19 patients, often associated with para-infectious processes, and potentially with direct infection within the CNS. Recent breakthroughs in the treatment of coronaviral infections in cats, including neurological FIP, have utilized antiviral drugs similar to those currently in human COVID-19 clinical trials. Differences in specific coronavirus and host factors are reflected in major variations in incidence and mechanisms of CNS coronaviral infection and pathology between species; however, broad lessons relating to treatment of coronavirus infection present within the CNS may be informative across species.

## Introduction

The Coronaviridae family of viruses are single-stranded RNA viruses found in a variety of species including cats, dogs, horses, mice, birds, pigs, bats, camels, whales, and humans (1). Coronaviruses are grouped into four genera; alpha, beta, gamma, and delta (Table 1) and viral particles contain four main structural proteins, namely, spike (S), envelope (E), membrane (M), and nucleocapsid (N) with specific coronaviruses also having a unique set of accessory proteins (2, 3). The distinctive trimeric spike protein (S) is primarily responsible for recognition of cellular receptors associated with viral binding and potentially internalization of host target cells (2–4). Many key receptors interacting with the spike proteins have been defined for the known coronaviruses (Table 1).

**Table 1:** Coronaviruses of humans and domestic animals.

**Genera**	**Species**	**Virus**	**Disease association**	**Receptor**
**Alpha Coronavirus**	**Cat**	**FCoV Ser I**	Feline infectious peritonitis	Unknown
		**FCoV Ser II**	Feline infectious peritonitis	APN
	**Human**	HCoV-NL63	Respiratory disease, gastroenteritis	ACE2
		HCoV-229E	Respiratory disease	APN
	**Pig**	TGEV	Transmissible gastroenteritis	APN
		PEDV	Endemic diarrhea	APN
		CSeCoV, SADS-CoV	Diarrhea	Unknown
		PRCV	Respiratory disease	APN
	**Dog**	CECoV	Enteric disease	APN
**Beta Coronavirus**	**Human**	**SARS-CoV-2**	COVID-19	ACE2
		SARS-CoV	Severe acute respiratory syndrome	ACE2
		MERS-CoV	Middle East respiratory syndrome	DPP4
		HCoV-HKU1	Respiratory disease	Sialic acids
		HCoV-OC43	Respiratory disease	Sialic acids
	**Pig**	**Porcine hemagglutinating encephalitis virus (PHEV)**	Vomiting-wasting/encephalomyelitis	NCAM
	**Mouse**	**Murine hepatitis virus (MHV)**	Hepatitis/encephalitis	CEACAM1
	**Cow**	BCoV	Enzootic pneumonia/Diarrhea-enteritis	Sialic acids
	**Dog**	CRCoV	Respiratory disease	Sialic acids
**Gamma Coronavirus**	**Avian**	IBV	Infectious bronchitis	α-2, 3-Linked sialic acid
**Delta Coronavirus**	**Pig**	PDCoV/PCoV-HKU15	Diarrhea	APN

*APN, aminopeptidase N; ACE2, angiotensin-converting enzyme 2; DPP4, dipeptidyl peptidase-4; NCAM, neural cell adhesion molecule; CEACAM1, carcinoembryonic antigen-related cell adhesion molecule 1. Viruses in red represent coronavirus disease commonly presenting with neurological signs*.

Coronavirus infections typically affect the respiratory or gastrointestinal tracts; however, coronavirus-related neurological disease is receiving increased attention as the COVID-19 (SARS-CoV-2) pandemic progresses. Neurological manifestations of COVID-19 infections in humans have become more widely recognized as a significant component of clinical disease (5–16); however, coronavirus involvement of the nervous system is not unique to the SARS-CoV-2.

Several coronaviruses have been associated with neurological disease as a common clinical presentation (Table 1), including feline infectious peritonitis (FIP), porcine hemagglutinating encephalitis virus, murine hepatitis virus (MHV), and currently with SARS-CoV2 virus in COVID-19 patients. Less commonly, the human respiratory disease coronaviruses HCoV-299E and OC43 have been demonstrated in brains of multiple sclerosis (17–21) and encephalitis patients (22–24). Coronavirus-associated encephalitis has been reported in children (25), and sporadic neurological disease has been reported in human Middle Eastern Respiratory syndrome (MERS) and SARS-CoV patients (26–33) although in a relatively limited manner compared to SARS-CoV-2 patients (27, 28, 34).

## Mechanism of CNS Entry

Several mechanisms of entry of coronaviruses into the CNS have been postulated and vary depending on the specific coronavirus, host factors, viral dose, and site of infection. Mechanisms are incompletely or poorly understood in many species; however, hematogenous spread via capillary endothelial cells, retrograde axonal transport via olfactory, pulmonary vagal and enteric neurons, exosomes, and entry via macrophage/monocytic cells have been suggested as potential mechanisms (35–41). Porcine hemagglutinating encephalitis virus has been shown to infect the CNS via retrograde transport in peripheral nerves from primary sites of replication (40, 42), and a similar mechanism of entry has been shown for neurotropic strains of MHV (43) and in a SARS mouse disease model (37).

S protein interaction with cell surface receptors (Table 1) is a major determinant of virus virulence and tropism allowing cell binding; subsequent cleavage of the bound spike protein by cellular proteases such as transmembrane serine protease 2 (TMPRSS2) allows internalization by direct fusion with the plasma membrane or use of endocytic mechanisms. Specific coronavirus target receptors have been shown to be variably expressed in a variety of infected CNS cell types (36, 44, 45); however, virus–host interactions are complex as not all infected cells necessarily express a single receptor, additional mechanisms such as receptor independent fusion can occur (46), and binding and entry may utilize similar or different receptors for some viruses (47). Major receptors for the CNS-tropic coronaviruses have been defined in most species, including angiotensin-converting enzyme 2 (ACE2) utilized by human coronaviruses HCoV-NL63, SARS-CoV, and SARS-CoV-2; however, the specific mechanism by which the pre-dominant Type I pathogenic feline coronaviruses attach and enter host cells is poorly defined (48–50).

## Mechanism of CNS Disease

Viral-mediated CNS damage may arise due to direct effects of viral replication within target cells and as a consequence of the vigorous inflammatory response that may have both positive anti-viral and potentially negative secondary effects (51, 52). Profound activation of inflammatory and immune cascades driven by a variety of cytokines and chemokines, including IL6, CXCL10, IL1, IFNγ, and TNFα have been documented in CNS coronavirus infections in a variety of species (11, 25, 37, 51, 53–55). Secondary immune-mediated mechanisms of pathology have also been described relating to the presence of viral antigens and antibody-mediated type III hypersensitivity vasculitis (56, 57). Although poorly defined, coronavirus CNS infections may also result in more chronic disease, as is seen with some strains of MHV (51, 56), and human coronavirus infection has been implicated in the pathogenesis of chronic conditions including Parkinson's disease, multiple sclerosis, and peripheral neuropathies (7, 12, 17, 19, 58).

Clinical and pathological findings in the most commonly affected species with CNS-associated coronavirus diseases is quite variable and likely reflects the variability in cellular tropism, mechanism of infection, and-immune mediated characteristics of disease in the different species. Para-infectious mechanisms, with neurological consequences secondary to extra-CNS disease factors such as sepsis and vascular disease, may also be important when the CNS is not the primary target organ as is the case for COVID-19 patients with acute respiratory disease (5, 7, 8, 59, 60).

### Feline Infectious Peritonitis

Feline infectious peritonitis virus is a pathotype of the feline enteric coronavirus (FECV) arising through specific mutations in key viral genes [reviewed in (38)]. Feline infectious peritonitis is named for the more commonly presenting effusive “wet” form of the disease, with a less common “dry” form characterized by granulomatous disease in the absence of marked inflammatory exudation into body cavities (57). Both FECV and FIP biotypes exit as one of two serotypes (61, 62). Type I is the more common serotype and possibly more likely to cause disease (62–64), while type II represents a recombinant between feline and canine enteric coronaviruses (65). Neurological involvement with FIP is well-documented (57, 66–70), occurs in ~30–40% of cats presenting with the non-effusive form of the disease (57), and is almost universally fatal (57).

Coronavirus infections resulting in FIP do not generally infect primary CNS cells. Pathogenic transformation of the FECV to the FIP biotype involves a marked alteration of tropism from apical epithelial enteric cells to internalization and replication within macrophages/monocytes (71, 72) that pre-dominantly represents the infected cell population within the CNS. Histopathology reflects the pre-dominant immune-mediated perivasculitis mechanism of disease with a lymphoplasmacytic infiltrate and variable presence of macrophages and neutrophils, often perivascular and typically centered around the leptomeninges and ependyma. Lesions particularly affect the caudal brainstem with perivascular oriented meningitis, periventricular and superficial encephalitis, and choroiditis with secondary hydrocephalus (57, 66, 67, 70).

### Mouse Hepatitis Virus

Unlike FIP virus, MHV is capable of infecting ependymal cells, astrocytes, microglia, oligodendrocytes, and neurons (56, 73). Depending on specific virus and mouse strain as well as route of infection, a variety of neuropathologies are seen with MHV infection, from acute encephalitis to a more chronic encephalomyelitis and demyelinating disease (56). Mixed inflammation with a significant neutrophilic component is typically present often centered around the choroid plexus, ependyma, and meninges (51, 74, 75).

### Porcine Hemagglutinating Encephalomyelitis Virus

In contrast to MHV, PEHV causes a non-suppurative encephalomyelitis with lymphoplasmacytic cuffing involving the gray matter of the cerebrum and neuronal degeneration of the brainstem and trigeminal ganglia (42). Viral infection is restricted to the neuronal perikaryon following spread from primary sites of replication via the peripheral nervous system (40, 76).

### Human CNS Coronavirus Infection

Detailed reports of cell tropism and histopathological lesions in human patients with coronavirus-associated neurological disease are lacking. SARS-CoV and HCoV-OC43 have been reported in cerebral neurons from autopsy specimens using immunohistochemistry and *in situ* hybridization (23, 32, 33, 77), and coronavirus has been similarly reported in unspecified cells from MS patients (17, 20). Neuronal degeneration, gliosis, and cerebral edema were the most consistent findings reported in SARS patients where histopathology of the brain was described (32, 33) and involvement of brainstem neurons has been proposed as a component of respiratory failure seen in patients (78, 79). Findings in COVID-19 patients are limited and variable. The most common underlying mechanisms of CNS involvement in COVID-19 patients remain to be defined (10, 80, 81), and direct evidence of virus in the CNS is limited. However, SARS-CoV-2 virus has been demonstrated specifically in the CSF (6, 80, 82, 83) and in brain tissue in up to 36% of COVID-19 patients examined at autopsy (59, 60, 84, 85). Variable neuropathological findings have been reported including subcortical white matter vascular and demyelinating lesions (86), lymphocytic meningoencephalitis with prominent neuronal loss (79), and hypoxic injury (60). Neuroimaging with MRI in 37 patients was similarly variable with common findings including signal abnormalities in the medial temporal lobe, multifocal white matter hyperintensities, and extensive white matter microhemorrhages (80).

Clinical neurological signs associated with the COVID-19 SARS-CoV-2 virus are variable and have been commonly associated with sequelae secondary to systemic effects of COVID-19 infection as well as primary viral effects on the CNS and peripheral nervous system. Common presentations include encephalopathy with delirium/psychosis, inflammatory CNS syndromes, ischemic strokes, peripheral neurological disorders including Guillain–Barre syndrome, and an/hyposmia and dys/hypogeusia (altered sense of smell and taste) (5, 6, 8–11, 13–16). As with other CNS coronaviral infections, the proposed pathological mechanisms include secondary inflammatory syndromes, secondary immune-mediated syndromes, neurological consequences of systemic disease including sepsis, hypoxia, and hypercoagulability, and direct neuronal/glial cell injury.

## Treatment

Data relating specifically to treatment of naturally occurring CNS coronavirus infections is extremely limited in humans, domestic, and production animals. Therapeutic approaches are generally similar regardless of organ systems affected; however specific issues relating to the blood–brain barrier/blood–CSF barrier limitations on drug delivery and pronounced neurological effects due to secondary inflammation need to be considered. The variable pathogenesis and clinical aspects of coronavirus disease in non-human species means that translational therapeutic studies in these animals may have some limitations. However, CNS coronaviral infections in domestic cats (FIP), in particular, may be translationally valuable given both the severity of disease presentation and the individualized approach to treatment in a companion vs. production or research setting. Recent data relating to treatment of both non-CNS and CNS FIP with antiviral drugs may have relevance to specific aspects of ongoing trials in SARS-CoV-2 patients. Interestingly, domestic and big cats are susceptible to SARS-CoV-2 infection, consistent with expression of ACE2 viral receptor in these species (87, 88), although associated clinical CNS disease has not been reported (89, 90).

Management of coronavirus infections consists of a variety of preventative and therapeutic approaches based on pathogenic mechanisms of the targeted coronaviruses as well as species-specific aspects of clinical disease. Several reviews of therapeutic aspects of coronavirus infections are available and discuss the main arms of disease management relating to prevention, husbandry, vaccination, antiviral drugs, and modulation of immune/inflammatory aspects of coronavirus infections in humans (91–94) and domestic animals (57, 95–98).

### Preventative

Preventative management, beyond husbandry, and environmental management of disease outbreaks is centered around vaccination. The value of vaccination depends on both severity of the disease and efficacy/longevity of the vaccines developed. Development of effective vaccines for human coronavirus infections, particularly SARS-CoV-2 (COVID-19), is an ongoing priority (91, 92). Inactivated and live attenuated vaccines have been shown to provide protective immunity in several domestic species (98); however, the value of vaccination has to be balanced against expense and prevalence of disease. Immunological sequelae following coronavirus infection appears to play a major role in disease progression, particularly in the CNS, and adverse events associated with vaccination must be considered in this context. Immunity to FIP is largely cell mediated, and humoral immunity with systemic antibodies to FIP virus may exacerbate disease by enhancing viral uptake and replication in macrophages and by stimulating a vascular-oriented Arthus-type hypersensitivity reaction (57, 99). An intranasal temperature-sensitive mutant FIP vaccine generating a local IgA response has been shown to have efficacy; however, its value in the clinical setting is questionable (57).

### Anti-inflammatory/Immunomodulatory Therapies

Dexamethasone is one of the few therapies that has been shown to have a beneficial effect in COVID-19 patients (100), although the pros and cons of anti-inflammatory vs. immunosuppressive effects have been debated with COVID 19 as with other coronaviruses such as SARS-CoV and MERS-CoV. Use of corticosteroids and intravenous immunoglobulin therapy for non-specific inflammatory and potential immune-mediated aspects of CNS disease have been anecdotally reported in neurological COVID-19 (8). Non-specific anti-inflammatory drugs such as corticosteroids, cyclophosphamide, and cyclosporine have anecdotally been associated with amelioration of signs in FIP CNS disease but are not curative (57, 66, 95–97). More targeted inhibition of specific cytokines such as TNFα have shown mixed therapeutic benefits in systemic FIP (101–103), and poor responses have generally been seen with the use of interferons α, β, and omega (57, 96, 97).

### Antivirals—Lessons From Feline Trials

A wide spectrum of antiviral drugs has been developed targeting most aspects of the coronavirus life cycle [reviewed in (92)], including neutralizing antibodies (convalescent plasma or monoclonal), fusion and viral protease inhibitors, nucleoside analogs, host protease and receptor inhibitors, and lipidomic reprogramming drugs. The nucleoside analogs ribavirin, NHC (β-D-N4-hydroxycytidine), and remdesivir/GS-5734 have activity against a variety of RNA viruses including coronaviruses. Chloroquine/hydroxychloroquine is an antimalarial and autoimmune drug that can block viral infection by increasing endosomal pH (required for virus-cell fusion) and can also interfere with glycosylation of cellular receptors. Remdesivir and chloroquine can inhibit SARS-CoV-2 *in vitro* (104) and are in trials for COVID-19 patients. There is currently no evidence for a beneficial effect of chloroquine/hydroxychloroquine in COVID-19 patients (105), and chloroquine had only modest effects in cats with experimentally induced FIP, and toxicity with elevations of serum alanine aminotransferase has been noted (106).

**Figure 1:**
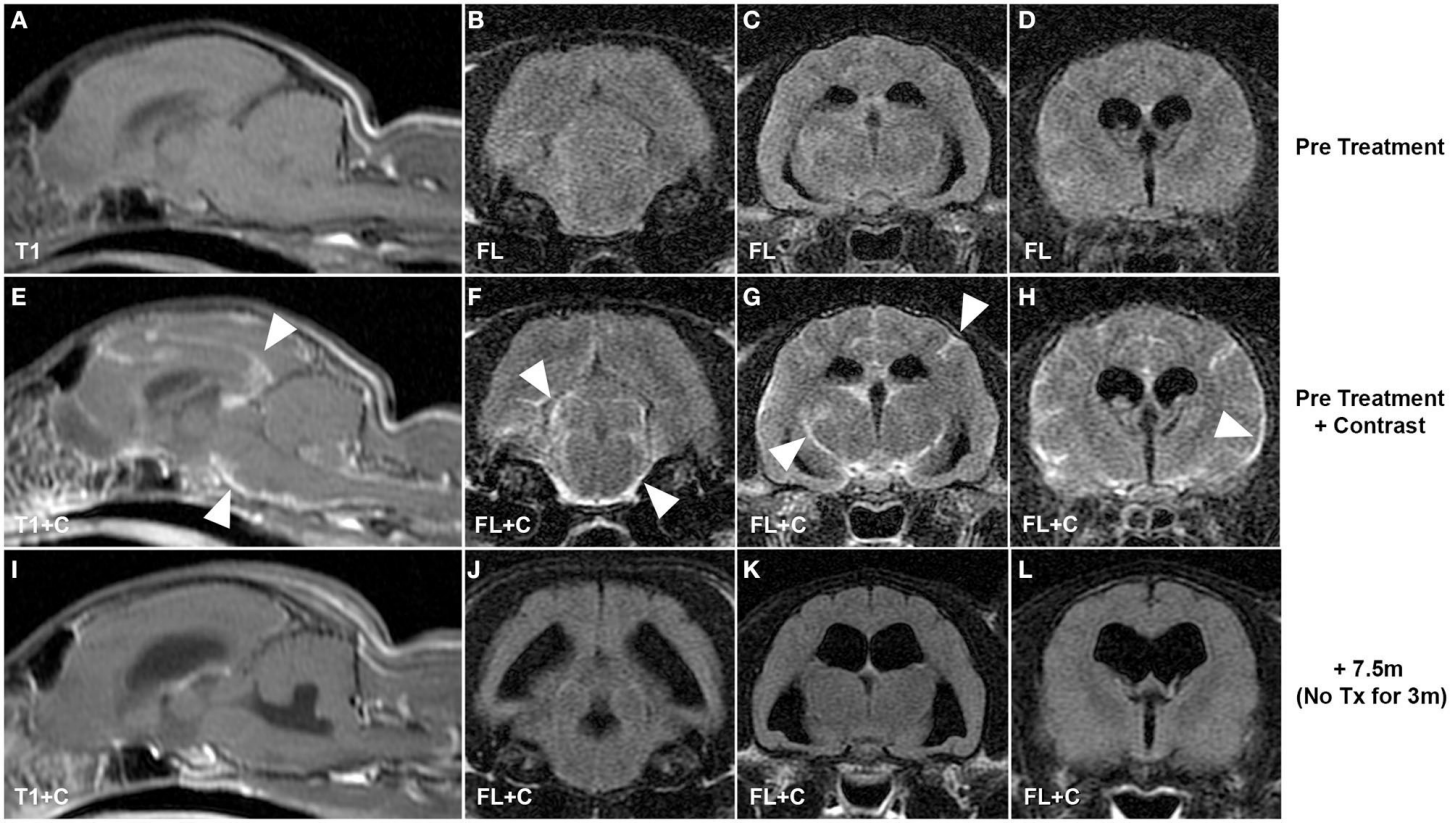
CNS coronavirus infection (FIP) in a cat presenting with neurological deficits and treated with GS-441524, the parent nucleoside of remdesivir. Pre-contrast **(A–D)** and post-contrast T1-weighted and fluid-attenuated inversion recovery pre-treatment MRI sequences **(E–H)** reveal multifocal leptomeningeal lesions (arrowheads) typical of CNS FIP. Resolution of clinical signs was incomplete using drug dosing typically effective in non-CNS disease (4 mg/kg); however, increased dosing (10 mg/kg) resulted in resolution of clinical signs and resolution of MR lesions on images acquired 7.5 months after initiation of treatment and 3 months after completion of treatment **(I–L)**. T1, T1-weighted; FL, fluid-attenuated inversion recovery; +C, contrast (gadopentetate dimeglumine, “Magnevist”).

Recent trials using antiviral drugs in clinical FIP have been extremely encouraging that treatment and potential cures are a realistic goal, including for CNS disease. Screening of large numbers of antiviral compounds to identify individual and combinations of drugs shows promise for future effective FIP therapies (107) and may address concerns relating to development of resistance with single drug regimens (108, 109). However, monotherapy with the nucleoside analog GS-441524 (Gilead Sciences Inc.) and a 3C-like antiviral protease inhibitor (Anivive Life Sciences Inc.) have already shown efficacy in experimental and naturally acquired non-CNS FIP (108, 110–112), although limitations associated with drug access across the blood–brain barrier resulted in CNS relapses, particularly with protease inhibitor therapy (108, 112). Cat pharmacokinetic data for GS-441524 showed that CSF concentrations of GS-441524 were ~20% of plasma levels (111) and that doses five times those shown to effectively treat non-CNS FIP (2–4 mg/kg) would be necessary to achieve 1 μM concentrations consistent with the *in vitro* 50% effective concentration (EC_50_) to prevent coronavirus cytopathic effects. Subsequent pilot data from cats presenting with CNS FIP supported these data with resolution of disease signs and apparent cures with dosing up to 10 mg/kg (Figure 1) (113). GS-441524 is a 1′-cyano-substituted adenine C-nucleoside ribose analog that inhibits viral RNA synthesis once it has been tri-phosphorylated intracellularly. Remdesivir (GS-5734) is a monophosphate prodrug of GS-441524 with the phosphate masked by McGuigan prodrug moieties designed to promote release of the monophosphorylated analog intracellularly and to overcome the perceived rate-limiting first phosphorylation step. Remdesivir has been given emergency use authorization for treatment of SARS-CoV-2 with encouraging if limited preliminary results (114–116). Given the efficacy of GS-441524 in the treatment of FIP, it has been suggested that there may be advantages to the use of the parent (GS-441524), rather than the prodrug (remdesivir) in human trials (117). Remdesivir appears to be rapidly metabolized in the serum to GS-441524 rather than entering cells intact (118, 119), and GS-441524 can be present in the serum at concentrations 1,000-fold higher than remdesivir (118). *In vitro* comparison of antiviral efficacy of remdesivir and GS-441524 against SARS-CoV and MERS-CoV showed similar EC_50_ values, and GS-441524 values were lower in some cases than the EC_50_ values reported in feline CRFK cells (Crandel Reese Feline Kidney Cells) infected with FIP virus (109, 111). GS-441524 serum levels in humans would more likely exceed these EC_50_ values based on published data (117), and similarities to cat *in vitro* data together with the encouraging clinical efficacy in cat FIP (111–113) would support the investigation of GS-441524 for use in human coronaviral disease, including CNS infections. Current dosing of remdesivir in COVID-19 trials is 200 mg loading followed by 100 mg (114, 115), equivalent to 1.5–3 mg/kg for a 70-kg human. These doses fall within the range shown to be effective in treating non-CNS FIP in cats (111, 112); however, the increased doses necessary to treat CNS FIP infections (8–10 mg/kg) in cats (113) would be equivalent to 560–700 mg for a 70-kg human. GS-441524 appears to have a high therapeutic index and minimal adverse effects at all doses of GS441524 reported in cats (2–10 mg/kg) (111–113). CNS blood–brain, blood–CSF barrier pharmacokinetic limitations are likely to be similar between cats and humans, and experience with FIP suggests that dose escalation of remdesivir (or GS-441524) may be necessary to optimize clinical efficacy in humans if targeting of coronavirus within the CNS is a specific therapeutic goal.

GS-441524 is not approved or available for clinical veterinary use limiting the potential for expanded and regulated clinical studies necessary to support approval in clinical veterinary practice. Unapproved sources of GS-441524 have become available online to owners of FIP cats, and FIP advocacy groups have collated observational data relating to outcomes in these “owner-treated” animal cohorts. Data arising from unverified drug sources and owner reported outcomes have major limitations; however, against a historical background of almost universal fatality in cases of CNS FIP, some clinically relevant data may be available. Advocacy group treatment regimens, based on published data (111–113), typically recommend a minimum 12-week course of treatment, with 4- to 6-mg/kg doses for non-CNS FIP treatment and 8- to10-mg/kg doses for CNS disease cases. Cure or remission is defined as no evidence of clinical disease >12 or <12 weeks, respectively, after completion of treatment. Data from an FIP advocacy group (personal communication) detailing owner outcomes from 110 cats with neurological signs and presumptive FIP treated with unapproved GS-441524 drug showed the following: 57/110 (52%) in remission, 22/110 (20%) cured, 9/110 (8%) died or euthanized, and 7/110 (6%) with relapsed CNS disease. Fifteen cats (14%) presented with non-CNS disease but relapsed with CNS signs following treatment. Sequential dose data was available for five cats that relapsed with CNS disease; initial doses ranged from 5 to 7 mg/kg, and four of five cats were subsequently cured with one in remission following dose escalation to 10–16 mg/kg. These uncontrolled data are supportive of the efficacy previously documented in four cats treated with GS-441524 (113) and of the necessity of increased dosing for optimal treatment of CNS infections. A striking aspect of GS-441524 treatment of FIP is the dramatic (often <36 h) improvement in clinical signs following adequate dosing (112, 113). Resolution of gross neuropathology in this time period is unlikely, and it is possible that decreased production of inflammatory cytokines, known to be a significant component of CNS coronaviral pathology, may be responsible for this rapid clinical improvement. Whether similar clinical correlates will be present with treatment of human coronaviral infections with GS-441524 or remdesivir remains to be seen.

Naturally occurring coronaviral infections in companion and production animals have many similarities to human pandemic-related diseases such as SARS, MERS, and COVID-19, although species and virus-specific factors described above mean that broad translation of therapeutic data across species will have major limitations. However, findings relating to basic treatment-related factors such as blood–brain barrier effects on therapeutic drug penetration to the CNS are likely to be relevant across species. It is currently unclear to what degree viral infection of the CNS impacts the clinical outcome in COVID-19 patients and how it may influence therapeutic practice; however, advances in the treatment of previously fatal coronavirus infections in cats with antiviral nucleoside analog drugs, particularly in the context of CNS infection, is encouraging that similar approaches may be efficacious in other species.

## Data Availability Statement

The raw data supporting the conclusions of this article will be made available by the authors, without undue reservation.

## Author Contributions

PD conceived and wrote the manuscript.

## Conflict of Interest

The author declares that the research was conducted in the absence of any commercial or financial relationships that could be construed as a potential conflict of interest.
